# S01-3 Determining the social and economic value of football in England

**DOI:** 10.1093/eurpub/ckac093.004

**Published:** 2022-08-29

**Authors:** Lottie Birdsall-Strong

**Affiliations:** The Football Association, England, London, United Kingdom

**Keywords:** Social value, Football, Team sport

## Abstract

**Background:**

In 2015 the Department of Culture, Media and Sport in England set out its new guidelines to redefine what success in sport means. This new focus on the social impact of sport including physical wellbeing, mental wellbeing, individual development and social and community development, meant that any funding decisions will be made based on the outcomes that the sporting body can deliver. The Football Association have therefore begun a detailed assessment of the social and economic value of football in England.

**Methods:**

Using national participation surveys of approximately 9000 individuals aged 16-75, we calculated the association between regular football participation and several health and social outcomes. This was combined with a review of published studies through to December 2019 examining the relationship between football, physical activity and disease risk outcomes across the life-course. This evidence base was then modelled with the estimated national football participation rate and the latest figures on national disease prevalence across 10 different diseases, to quantify the true value of football to society.

**Results:**

Preliminary results have shown that football participation contributes to more than £10 billion to society each year across England. Team sport participants also reported significantly higher levels of happiness, confidence and trust compared to individuals who played no sport, or just individual sport. This impact also varies depending on the demographic groups including women, children and low-socioeconomic groups.

**Conclusions:**

This analysis shows the large contribution of grassroots football to the nation's economy and to health and social wellbeing of individuals and communities. Moreover, it has proven the unique benefits of team sport participation and shown to policymakers which type of sports and activities contribute the most value to society.

